# Phenotypic and genetically predicted leukocyte telomere length and prostate cancer risk: results from a large-scale longitudinal cohort study

**DOI:** 10.7189/jogh.15.04228

**Published:** 2025-09-26

**Authors:** Xiaoyang Liu, Shengzhuo Liu, Yunfei Yu, Pan Song, Luchen Yang, Zhenghuan Liu, Jing Zhou, Xin Yan, Kai Ma, Haiyun Qiu, Xianding Wang, Qiang Dong

**Affiliations:** 1 Department of Urology, Institute of Urology, West China Hospital, Sichuan University, Chengdu, China; 2 Kidney Transplantation Center, West China Hospital, Sichuan University, Chengdu, China

## Abstract

**Background:**

Previous studies on the correlation between leukocyte telomere length (LTL) and prostate cancer (PCa) have shown inconsistent results. We aimed to clarify this association by leveraging a large-scale prospective design and Mendelian randomisation.

**Methods:**

We enrolled a total of 229 022 male individuals from the UK Biobank (UKB) to investigate the association between LTL and PCa risk. We employed both unadjusted and covariates-adjusted Cox proportional hazards regression models to assess this relationship. We defined the primary outcome as the diagnosis of incident PCa using in-patient data and the death registry of the UK Biobank cohort. To validate the reliability of the primary findings, we conducted secondary analyses, including Mendelian randomisation.

**Results:**

The primary analysis demonstrated that longer LTL was substantially associated with higher risk of PCa, with associations remaining robust after adjusting for potential covariates (hazard ratio (HR) = 1.444; 95% confidence interval (CI) = 1.247, 1.673, *P* < 0.001). We observed similar results when LTL was analysed as both a continuous and categorical variable, and the association was shown to be inversely U-shaped. We further validated the association at the genetic level using Mendelian randomisation across different PCa databases, with results consistent with our primary analysis.

**Conclusions:**

Our findings offer evidence that leukocyte telomere length is an important risk factor for PCa. Further studies are needed to elucidate the underlying mechanisms linking leukocyte telomere length to PCa risk.

Leukocyte telomere length (LTL) plays a crucial role in human biology, especially for preserving genomic integrity and shielding chromosomal ends from degradation [1]. Telomeres are repeated nucleotide sequences at the ends of chromosomes, and they progressively shorten with each cell division [2]. This process of shortening acts as a biological clock, signalling the age of cells and promoting their replicative senescence [3]. Due to the involvement in chromosomal instability and the ensuing cellular malfunction, shortened telomeres are linked to a range of age-related illnesses, such as cancer and cardiovascular diseases [4,5]. Telomere dynamics in cancer are complicated; both excessively short and abnormally long telomeres have been implicated in carcinogenesis, indicating a dual function of LTL in cancer initiation and progression [6,7].

Prostate cancer (PCa) is a major public health concern, especially for the ageing male population. It is one of the most often diagnosed malignancies in males and remains the primary cause of cancer-related deaths globally. The majority of PCa cases are identified in males over 50, and the frequency rises with age [8]. Comprehending the risk variables linked to PCa is vital to formulate focussed preventive measures and enhance outcomes for patients. The pathogenesis of PCa is still not fully known, despite improvements in diagnostic methods and treatment strategies. As a result, it is critical to find trustworthy biomarkers that may aid in risk assessment and early detection [9].

Shorter telomeres have historically been linked to chromosomal instability and higher cell turnover, making them a hallmark of cancer. Challenging this theory, emerging investigations point to a more complex link between telomere length and cancer risk [10]. There have also been contradictory findings from earlier research on the relationship between LTL and PCa risk [11–14]. Methodological limitations have hindered many of these investigations, including cross-sectional designs that make it impossible to infer causality, small sample sizes that introduce bias and lower statistical power, and a lack of genetic validation to support the reported associations [12,15].

Our work aims to provide a more thorough assessment of the relationship between LTL and PCa risk, addressing the existing deficiencies in the literature. By combining epidemiology analysis with Mendelian randomisation (MR), it seeks to provide new insights into the genetic and phenotypic underpinnings of LTL as a potential risk factor for PCa.

## METHODS

### Data resource and study population

**Figure 1:**
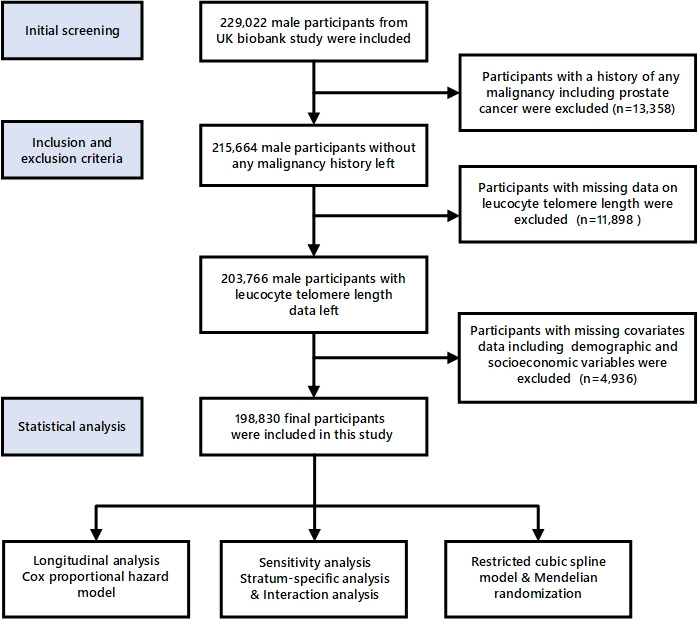
Flowchart of the total participants' selection and research design in the UK Biobank cohort.

The UK Biobank study is a population-based prospective investigation designed to collect data on the determinants of diseases prevalent during middle and older adulthood. Initiated between 2006 and 2010, approximately 500 000 men and women aged 40 to 69 years were recruited from various regions across England, Wales, and Scotland. They were drawn from among individuals registered with the UK's National Health Service. Further details on the protocols employed in the UK Biobank study can be accessed on their website [16]. Here we included a total of 229 022 male participants from the UK Biobank, from whom we excluded participants with a documented history of any malignancy, including PCa, at the time of recruitment (n = 13 358), those with missing data on leucocyte telomere length (n = 11 898), and those with missing covariates including demographic and socioeconomic variables (n = 4936) from the main analysis (**Figure 1**).

**Figure 2:**
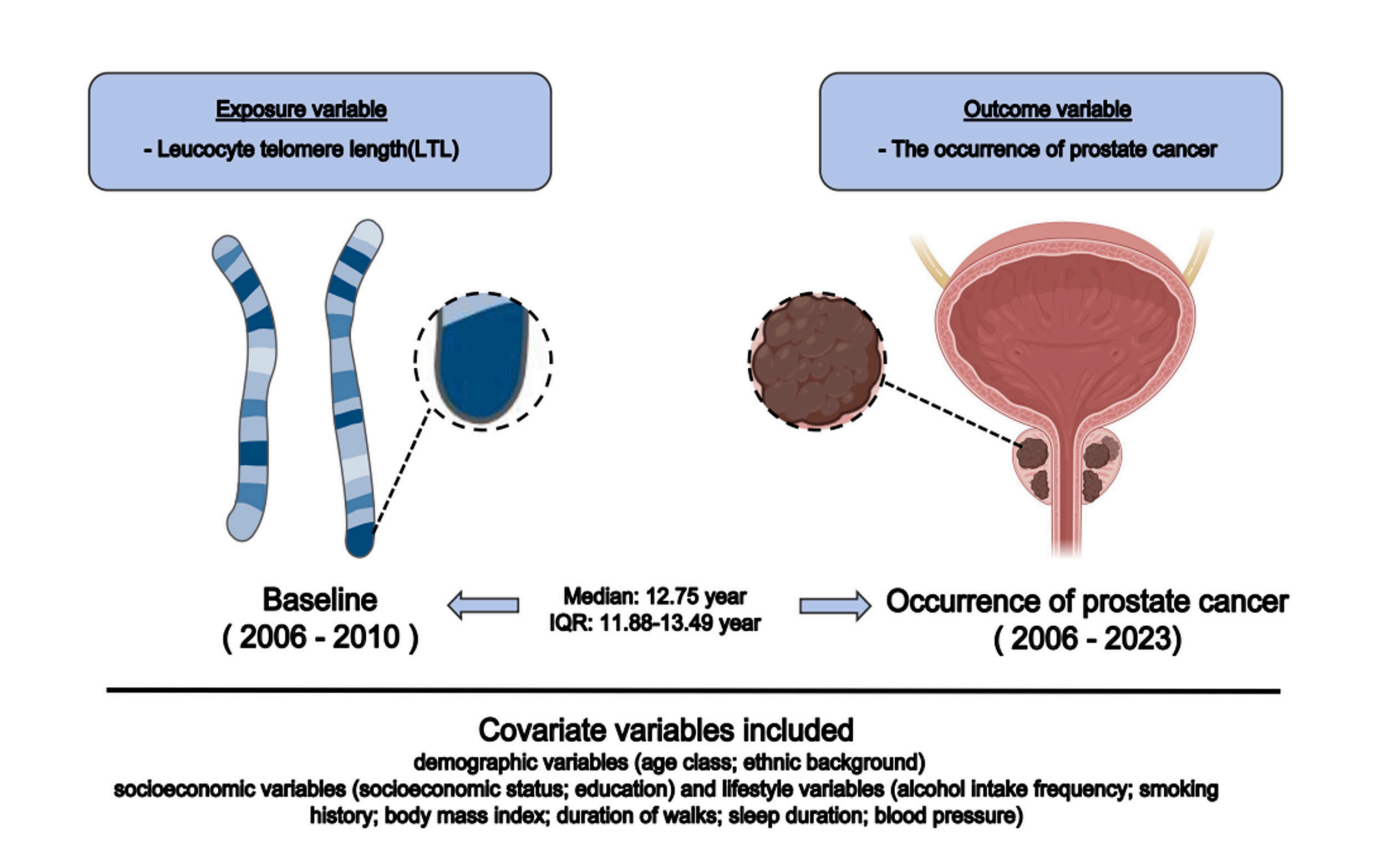
Graphical representation of the study design.

We assessed LTL during recruiting (baseline) and diagnosed PCa during the follow-up period following enrolment. We included demographic, socioeconomic, and lifestyle characteristics as baseline covariates (**Figure 2**).

### Definition of LTL measurements and malignant neoplasm of the prostate

The protocols for multiplex quantitative polymerase chain reaction (qPCR) test, quality control, and DNA extraction have all been previously documented [17]. The average telomere length of an individual’s leukocytes across all chromosomes is shown as the ratio of telomere (T) to single-copy gene copy number (T/S), which we further adjusted for batch variation, resulting in the adjusted relative T/S ratio [17]. Participants were diagnosed with PCa according to the International Classification of Diseases, 10th Revision codes C61, and were identified the first occurrence of PCa by mapping primary care data, hospital admissions data, death registry records, self-reported medical conditions, and other sources (UK Biobank Field 40005).

We formed the duration of the follow-up visits from the assessment centre attendance date (Field 53) to the first PCa diagnosis, date of death (Field 40000), or the last available date from the hospital inpatient data or primary care data [18].

### Covariates assessment

The UK Biobank cohort gathered demographic, medical, and lifestyle information through self-reported questionnaires, which covered various factors, including physical activity, sleep patterns, medication usage, alcohol consumption, and smoking history. Trained personnel directly measured participants' body mass index (BMI) and blood pressure, both automatically and manually, which was then classified according to the US Food & Drug Administration. We determined covariates selected as potential confounders based on their documented associations as reported in prior literature, and measured those that were relevant at baseline. Demographic variables included age (quantified in units of years and divided into three groups according to the boundary of 50 and 60) and ethnic background (White, Asian or Asian British, Black or Black British, mixed, and other ethnic group). Socioeconomic variables included socioeconomic status (quintiles of Townsend Index of deprivation) and education level (A levels/AS levels or equivalent, college or university degree, CSEs or equivalent, NVQ/HND/HNC or equivalent, O levels/GCSEs or equivalent, Other professional qualifications, none of the above). Lifestyle variables included alcohol intake frequency (never, <3 times/week, ≥3 times/week), smoking history (no/yes), and BMI (underweight, normal, overweight, and obesity, according to the cut-off values of 18.5, 25, and 30 kg/m^2^, respectively), duration of walks (0, 0–15, 15–30, 30–60, 60–180, or ≥180-minute/day), sleep duration (<7, 7–10, or ≥10 hours/day); blood pressure (normal, pre-hypertension, grade 1, grade 2, or grade 3). Multivariate imputation techniques were applied to address missing covariate data using predictive mean matching methods.

### Genetic summary data and Instrumental variables selection

We obtained data on genetic variants associated with LTL from recent research from the UK Biobank [19]. Circulating leukocytes provided the telomere DNA, and qPCR was used to determine LTL after age correction. For PCa, we collected the genome-wide association study (GWAS) summary data from the UK Biobank (9132 cases and 173 493 controls), the Prostate Cancer Association Group to Investigate Cancer-Associated Alterations (PRACTICAL) in the Genome Consortium (79 148 cases and 61 106 controls) and from the FinnGen Release 11 (17 258 cases and 143 624 controls) (Table S1 in the **Online Supplementary Document**).

### Statistical analysis

We used standard deviations (SD) and means (x̄) for continuous variables with a normal distribution and MDs with interquartile ranges for those with a skewed distribution. We used frequencies and percentages to present categorical variables. We used the Kruskal-Wallis test compare continuous variables with skewed distributions and χ^2^ tests to compare categorical data.

We calculated hazard ratios (HRs) and 95% confidence intervals (CIs) to examine the relationship between LTL and the risk of PCa. Cox proportional hazards regression models were utilised, with follow-up time as the time scale, defined from the baseline enrolment visit date until the occurrence of PCa diagnosis, date of death, loss to follow-up, or the end of the cohort's follow-up period, whichever event transpired first. We analysed LTL as continuous variables using multivariable Cox regression models, and we evaluated the proportional hazards assumptions using tests based on Schoenfeld residuals. We adjusted model I for age, model II for demographic variables (age, ethnic background) and socioeconomic variables (socioeconomic status, education), and model III for demographic variables (age class, ethnic background) and socioeconomic variables (socioeconomic status, education), and lifestyle variables (alcohol intake frequency, smoking history, BMI, duration of walks, sleep duration, blood pressure). We analysed LTL categorically, with the lowest quartile serving as the reference category. We also utilised restricted cubic spline models in conjunction with Cox proportional hazards models to examine potential nonlinear relationships, while adjusting for covariates as outlined in model III. The presence of potential nonlinearity was assessed using a likelihood ratio test, comparing the linear model against one that included both linear and cubic spline terms. Lastly, we used stratified analyses to assess potential modification effects based on all possible factors.

In MR analysis, a variety of weighting schemes, including inverse variance weighted (IVW), MR-Egger, weighted median, simple mode, and weighted mode, were used to examine the relationship between telomere and PCa [20]. We designate IVW as the primary outcome if these approaches provide different results. We determined the presence of horizontal pleiotropy using MR-Egger, with a *P*-value >0.05 indicating the absence of observable horizontal pleiotropy. We utilised Cochran's Q test to assess heterogeneity [21]. We used random-effects IVW models if there was heterogeneity, while the fixed-effect IVW model was used in other cases. Leave-one-out sensitivity analysis was used to assess the validity of the results and identify any outliers. We assessed the effectiveness using the F-statistic (F = R2 × (N-K-1) / (1 - R2)) (Figure S1 in the **Online Supplementary Document**) [22]. All findings were considered to be statistically significant at the level of 0.05, and we conducted all statistical analyses using *R*, version 4.2.2 (R Core Team, Vienna, Austria).

## RESULTS

### Baseline characteristics

After screening, 19 830 participants without any history of malignancy, including PCa, were included at baseline (**Table 1**). Over a median follow-up period of 12.75 years (interquartile range = 11.88–13.49 years), a total of 10 693 men were diagnosed with PCa. Compared to those without PCa, patients with PCa were older at enrolment and were more likely to have lower socioeconomic status, lower educational attainment, a history of smoking, reduced physical activity levels, and higher blood pressure.

**Table 1:** Baseline characteristics of the participants enrolled*

	Prostate cancer		
	**Yes (n = 10 693)**	**No (n = 188 137)**	***P*-value**
**Follow-up time in years, MD (IQR)**	6.87 (3.79, 9.46)	12.82 (12.07, 13.54)	<0.001
**Age when attended assessment centre, MD (IQR)**	62.00 (58.00, 66.00)	57.00 (49.00, 63.00)	<0.001
**Age class**			<0.001
<55	1441 (13.48)	76 228 (40.52)	
≥55	9252 (86.52)	111 909 (59.48)	
**Townsend, n (%)**			<0.001
≤−3.94	2462 (23.02)	37 690 (20.03)	
≤−2.77	2285 (21.37)	37 669 (20.02)	
≤−1.26	2240 (20.95)	37 756 (20.07)	
≤1.46	1995 (18.66)	37 726 (20.05)	
>1.46	1711 (16.00)	37 296 (19.82)	
**Qualifications**			<0.001
A levels/AS levels or equivalent	1062 (9.93)	19 733 (10.49)	
College or University degree	3601 (33.68)	64 531 (34.30)	
CSEs or equivalent	322 (3.01)	10 921 (5.80)	
None of the above	2123 (19.85)	31 945 (16.98)	
NVQ or HND or HNC or equivalent	1042 (9.74)	17 076 (9.08)	
O levels/GCSEs or equivalent	1944 (18.18)	35 607 (18.93)	
Other professional qualifications, *e.g.* nursing, teaching	599 (5.60)	8324 (4.42)	
**Current employment status**			<0.001
Doing unpaid or voluntary work	31 (0.29)	542 (0.29)	
Full or part-time student	17 (0.16)	391 (0.21)	
In paid employment or self-employed	5170 (48.35)	118 930 (63.21)	
Looking after home and/or family	35 (0.33)	1081 (0.57)	
Retired	5032 (47.06)	55 023 (29.25)	
Unable to work because of sickness or disability	253 (2.37)	7581 (4.03)	
Unemployed	155 (1.45)	4589 (2.44)	
**Ethnic background**			<0.001
Asian or Asian British	118 (1.10)	4882 (2.59)	
Black or Black British	215 (2.01)	2635 (1.40)	
Mixed	35 (0.33)	934 (0.50)	
Other ethnic group	65 (0.61)	1582 (0.84)	
White	10260 (95.95)	178 104 (94.67)	
**Smoking status**			0.009
No	5138 (48.05)	92 845 (49.35)	
Yes	5555 (51.95)	95 292 (50.65)	
**Alcohol intake frequency**			<0.001
Never	515 (4.82)	11 716 (6.23)	
<3 drinks/week	4190 (39.18)	79 544 (42.28)	
≥3 drinks/week	5988 (56.00)	96 877 (51.49)	
**BMI**			<0.001
Underweight	14 (0.13)	444 (0.24)	
Normal	2683 (25.09)	46 810 (24.88)	
Overweight	5581 (52.19)	92 763 (49.31)	
Obesity	2415 (22.58)	48 120 (25.58)	
**Duration of walks**			0.003
<15	883 (8.26)	16 313 (8.67)	
<30	2672 (24.99)	46 669 (24.81)	
<60	3188 (29.81)	54 752 (29.10)	
<180	3068 (28.69)	53 013 (28.18)	
≥180	882 (8.25)	17 390 (9.24)	
**Sleep duration**			<0.001
<7	2439 (22.81)	47 954 (25.49)	
<10	8048 (75.26)	137 017 (72.83)	
≥10	206 (1.93)	3166 (1.68)	
**Blood pressure**			<0.001
Normal	2488 (23.27)	52 290 (27.79)	
High-normal	2180 (20.39)	41 261 (21.93)	
Grade 1	4099 (38.33)	66 084 (35.13)	
Grade 2	1582 (14.79)	23 353 (12.41)	
Grade 3	344 (3.22)	5149 (2.74)	

IQR – interquartile range, MD – median

*Presented as n (%) unless specified otherwise.

### Associations between LTL and PCa

We initially treated LTL as a continuous variable (**Table 2**), with model I (which was adjusted for age) indicating that participants with longer LTL had a significantly higher likelihood of developing PCa (HR = 1.505; 95% CI = 1.301, 1.741; *P* < 0.001). This association remained significant in model II, which adjusted demographic (age, ethnic background) and socioeconomic variables (socioeconomic status, education) (HR = 1.454; 95% CI = 1.256, 1.683; *P* < 0.001). The relationship persisted in model III (HR = 1.444; 95% CI = 1.247, 1.673; *P* < 0.001) after further adjustment on model II for lifestyle factors (alcohol intake frequency, smoking history, BMI, duration of walks, sleep duration, blood pressure). We then analysed LTL categorically, with the lowest quartile (Q1) serving as the reference category. The analysis showed a nonlinear relationship when compared to the middle quartiles. In model I, the HRs were HR_quartile3_*_vs._*_quartile1_ = 1.090 (95% CI = 1.033, 1.151) and HR_quartile4_*_vs._*_quartile1_ = 1.187 (95% CI = 1.124, 1.253) (*P*-value for trend <0.001). This pattern remained consistent in Model II, with HR_quartile3_*_vs._*_quartile1_ = 1.086 (95% CI = 1.029, 1.146) and HR_quartile4_*_vs._*_quartile1_ = 1.174 (95% CI = 1.112, 1.240) (*P*-value for trend <0.001), and in model III, with HR_quartile3_*_vs._*_quartile1_ = 1.084 (95% CI = 1.028, 1.144) and HR_quartile4_*_vs._*_quartile1_ = 1.174 (95% CI = 1.108, 1.236) (*P*-value for trend <0.001). Finally, restricted cubic spline models fitted to Cox proportional hazards models demonstrated an inverse U-shaped connection (*P*-value for overall <0.001; *P*-value for nonlinearity = 0.018) between LTL and PCa risk (Figure S1 in the **Online Supplementary Document**).

**Table 2:** HRs and 95% CIs for the association between LTL (analysed both continuously and by quartile categories) and PCa risk in the UK Biobank study

				Model I*	Model II†	Model III‡
**Leukocyte telomere length**	**Cases**	**n**	**MD (IQR)**	**HR (95% CI)**	**HR (95% CI)**	**HR (95% CI)**
**Continuous**	10 693	198 830	0.810	1.505 (1.301, 1.741)	1.454 (1.256, 1.683)	1.444 (1.247, 1.673)
***P*-value for continuousness**				<0.001	<0.001	<0.001
**Q1 (0.170, 0.734)**	2761	49 708	0.683	ref	ref	ref
**Q2 (0.734, 0.810)**	2782	49 707	0.773	1.088 (1.032, 1.147)	1.087 (1.031, 1.145)	1.084 (1.029, 1.143)
**Q3 (0.810, 0.894)**	2607	49 707	0.849	1.090 (1.033, 1.151)	1.086 (1.029, 1.146)	1.084 (1.028, 1.144)
**Q4 (0.894, 5.364)**	2543	49 708	0.960	1.187 (1.124, 1.253)	1.174 (1.112, 1.240)	1.170 (1.108, 1.236)
***P*-value for trend**				<0.001	<0.001	<0.001

BMI – body mass index, CI – confidence interval, HR – hazard ratio, IQR – interquartile ranges, MD – median, ref – reference

*Adjusted for age.

†Adjusted for demographic variables (age, ethnic background) and socioeconomic variables (socioeconomic status, education).

‡Adjusted for demographic variables (age class, ethnic background), socioeconomic variables (socioeconomic status, education) and lifestyle variables (alcohol intake frequency, smoking history, BMI; duration of walks, sleep duration, blood pressure).

### Sensitivity analysis

**Figure 3:**
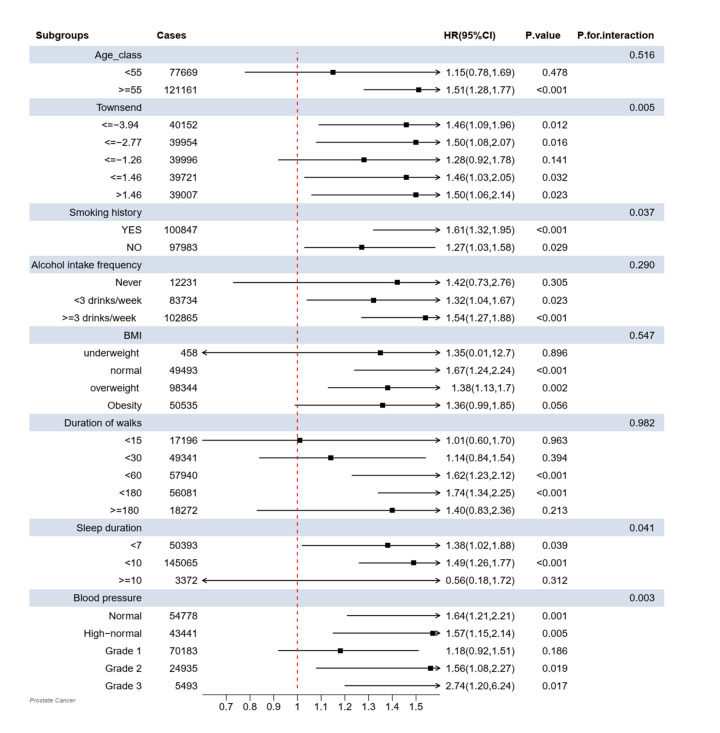
HRs and 95% CIs for the association between LTL and the risk of PCa in the UK Biobank cohort.

Building on the main analysis, we conducted subgroup analyses for LTL using model III (adjusted for demographic, socioeconomic, and lifestyle variables). The results for most subgroups (age class, alcohol intake frequency, BMI, duration of walks) were consistent with those observed in the entire cohort, with a few notable exceptions (**Figure 3**). Specifically, a significant association between LTL and PCa was observed among male participants across different Townsend deprivation index classes:≤−3.94 (HR = 1.46; 95% CI = 1.09, 1.96; *P* = 0.012);≤−2.77 (HR = 1.50; 95% CI = 1.08, 2.07; *P* = 0.016); ≤1.46 (HR = 1.46; 95% CI = 1.03, 2.05; *P* = 0.032); and >1.46 (HR = 1.50; 95% CI = 1.06, 2.14; *P* = 0.023), except for the≤−1.26 class. We observed a significant influence on PCa both in participants with a smoking history (HR = 1.61; 95% CI = 1.32, 1.95; *P* < 0.001) and those without (HR = 1.27; 95% CI = 1.03, 1.58; *P* = 0.029). Similarly, the association remained significant across different sleep durations: <7 hours (HR = 1.38; 95% CI = 1.02, 1.88; *P* = 0.039) and <10 hours (HR = 1.49; 95% CI = 1.26, 1.77; *P* < 0.001). This significant association was also observed in participants with normal (HR = 1.64; 95% CI = 1.21, 2.21; *P* = 0.001), high-normal (HR = 1.57; 95% CI = 1.15, 2.14; *P* = 0.005), Grade 2 (HR = 1.56; 95% C = 1.08, 2.27; *P* = 0.019), and Grade 3 (HR = 2.74; 95% CI = 1.20, 6.24; *P* = 0.017) blood pressure categories.

### Genetically predicted LTL on PCa in MR

Following the selection process, we identified a total of 138 single-nucleotide polymorphisms (SNPs) as genetic instruments for LTL. We obtained and evaluated PCa genetic instruments independently from the UK Biobank, the PRACTICAL collaboration, and the FinnGen. The primary analysis showed that genetically predicted longer LTL was linked to an increased risk of PCa in the UK Biobank (IVW: OR = 1.313; 95% CI = 1.163, 1.483, *P* < 0.001), the PRACTICAL consortium (IVW: OR = 1.024; 95% CI = 1.016, 1.032; *P* < 0.001), and the FinnGen (IVW: OR = 1.435; 95% CI = 1.173, 1.755; *P* < 0.001) (Figure S2, Panels A–C in the **Online Supplementary Document**). The results were consistent across several methodologies. The scatter plot and funnel plots further confirmed a positive association between longer LTL and increased PCa risk (Figures S2 and S3 in the **Online Supplementary Document**). The leave-one-out analysis did not find any specific SNP with a significant impact on the association (Figure S4 in the **Online Supplementary Document**).

## DISCUSSION

Here we noted a strong positive correlation between the risk of PCa and LTL. Our results consistently show that individuals with longer LTL are associated with an increased risk of PCa (HR = 1.444; 95% CI = 1.247, 1.673; *P* < 0.001). Moreover, we found an inverse U-shaped link between LTL and PCa risk (*P*-value for overall <0.001; *P*-value for nonlinearity = 0.018), and this correlation held across a variety of analytical techniques, including considering LTL as both a continuous and categorical variable. The MR and sensitivity analyses further supported the consistency of our findings.

Our findings offer a more sophisticated understanding compared to other studies on the connection between LTL and PCa risk, especially as previous research has produced contradictory findings related to various constraints. First, the majority of earlier research had small sample sizes, which raised the possibility of selection bias and limited the applicability of the results [13,23]. Second, the majority of studies were cross-sectional or case-control, which limited the capacity to establish a causal relationship and raised concerns about the reliability of statistical results [11,15]. Third, the absence of integration with genetic data has been a shortcoming in previous research and is critical to comprehending the hereditary aspects of telomere biology and its connection to cancer risk [11,12]. Additionally, most studies assumed a linear relationship between telomere length and cancer risk, neglecting the possibility of a nonlinear association. Our results indicate an inverse U-shaped association between LTL and PCa risk, suggesting that telomere length – both extremely short and very long – may be linked to an elevated risk of the disease [24]. This complexity emphasises the importance of considering nonlinear models when exploring the relationship between telomere length and cancer risk.

Although biological mechanisms underlying the connection between LTL and PCa have yet to be fully understood, several potential explanations can be considered. Telomeres preserve genomic integrity by protecting chromosomal ends from degradation and preventing end-to-end fusions [25]. Chromosomal instability has long been linked to short telomeres, and this instability can result in the accumulation of genetic abnormalities that cause cancer [26]. On the other hand, extended telomeres may offer a favourable environment for ongoing cell division, which might enable precancerous cells to evade replicative senescence and pick up new mutations that advance carcinogenesis [6]. Additionally, longer telomeres have been associated with a hyperproliferative state and enhanced survival of immune cells, which could modulate the tumour microenvironment in ways that promote cancer development [27]. Given telomere length's dual roles in cancer biology, maintaining an ideal telomere length is essential for both preventing cancer and preserving genomic stability. Our research has major implications for public health, especially in relation to PCa early identification and prevention. Given the correlation shown between longer LTL and higher PCa risk, LTL could serve as an effective biomarker for identifying high-risk patients and might help direct screening and monitoring initiatives. Further research could clarify the molecular pathways that connect LTL to PCa risk and investigate the possibility of telomere-targeted therapies. Such efforts should also concentrate on confirming these results in a wider range of individuals and examining the connection between LTL and other outcomes connected to cancer, such as survival and cancer progression.

Despite being one of the largest cohort studies to date investigating the genetic and phenotypic connection between LTL and PCa risk, our study does have certain limitations. First, the UK Biobank research sample is predominantly composed of individuals of European ancestry, which may restrict the applicability to other ethnic groups. Second, due to database limitations, there was insufficient information on the stage and progression of PCa, which precluded an analysis of the association between LTL and PCa development. The assessment of LTL samples may also introduce selection bias, since individuals in the UK Biobank are more health-conscious than the general population [15]. This ‛healthy volunteer’ bias could have potentially affected the observed associations.

## CONCLUSIONS

Previous studies examining the association between LTL and PCa have shown inconsistent findings. In this large-scale prospective cohort study, we demonstrate that both phenotypic and genetic telomere length are substantial risk factors for PCa. These findings provide new insights into the role of telomere biology in the development and progression of PCa.

## Additional material


Online Supplementary Document
