# Supplementary material for: Phenotypic and genetically predicted leukocyte telomere length and prostate cancer risk: results from a large-scale longitudinal cohort study
Source: J Glob Health. 2025 Sep 26;15:04228. doi: 10.7189/jogh.15.04228 (PMC12467445; doi:10.7189/jogh.15.04228)

Supplement to: Liu X, Liu S, Yu Y, Song P, Yang L, Liu Z, Jing Z, Xin Y, Ma K, Qiu H, Wang X, Dong Q. Phenotypic and genetically predicted leukocyte telomere length and prostate cancer risk: results from a large-scale longitudinal cohort study. J Glob Health. 2025;15:04228.

Phenotypic and genetically predicted leukocyte telomere length and prostate cancer risk: results from a large-scale longitudinal cohort study

[1] Department of Urology, Institute of Urology, West China Hospital, Sichuan University, Chengdu, China

[2] Kidney Transplantation Center, West China Hospital, Sichuan University, Chengdu, China

*** Correspondence:**
Xianding Wang; Qiang Dong
E-mail: xiandingwang@scu.edu.cn (WX). dongqiang@scu.edu.cn (DQ)

Table S1. selection of instrumental variables for the Mendelian Randomization (MR) analysis and data sources included in the MR analysis

Instrumental Variables (IVs) Selection Criteria	
Criteria 1	There must be a robust relationship between the exposures variables and the IVs selected for analysis. To attain a more comprehensive result, we chose IVs with the locus-wide significance level
Criteria 2	The IVs are required to pass the independence test. By excluding the SNPs using the PLINK clustering method (r2 > 0.001 and clump window 10,000 kb), the effect of linkage disequilibrium (LD) among the included genetic variants was avoided.
Criteria 3	Any IVs with a MAF value < 0.01 are excluded. Lastly, During the harmonization procedure, we eliminated palindromic SNPs to guarantee that SNP effects on exposure are associated with the same allele as SNP effects on outcom
Data sources included in the MR analysis	
Telomere length	
PCA of R11 release of the Finngen study	https://www.finngen.fi/en
PCA of Prostate Cancer Association Group to Investigate Cancer-Associated Alterations in the Genome (PRACTICAL)	PRACTICAL Consortium
PCA of UK biobank (ieu-b-4809)	https://gwas.mrcieu.ac.uk/

Supplementary Figure S1

Restricted cubic spline models fitted for Cox proportional hazards models for LTL and risk of PCa.

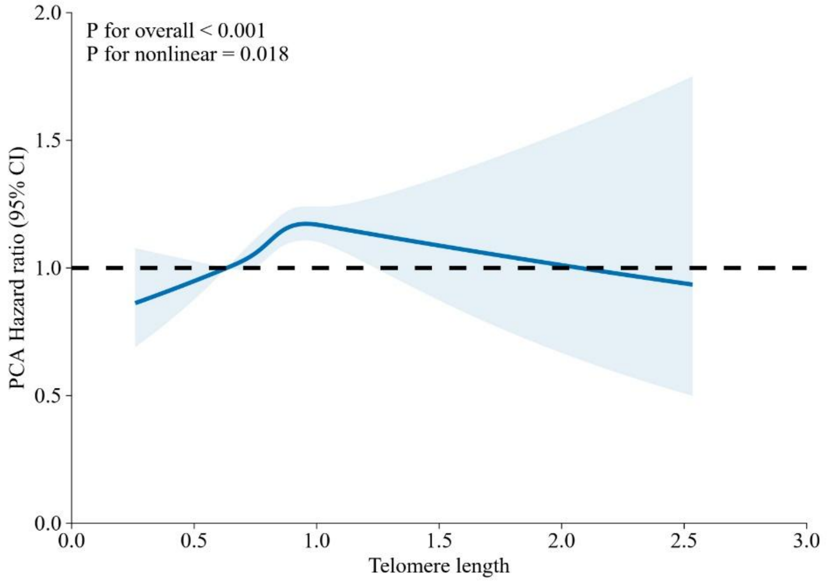

Supplementary Figure S2

The primary MR analysis of association between LTL and Pca (Figure a, UK Biobank; Figure b the PRACTICAL consortium; Figure c FinnGen study).

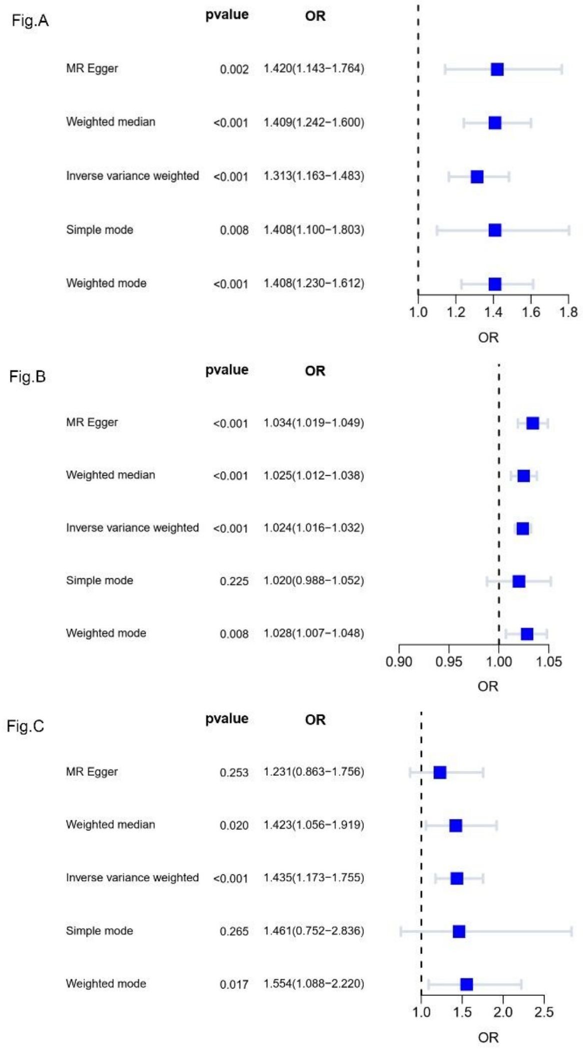

Supplementary Figure S3

Flowchart of the Mendelian randomization.

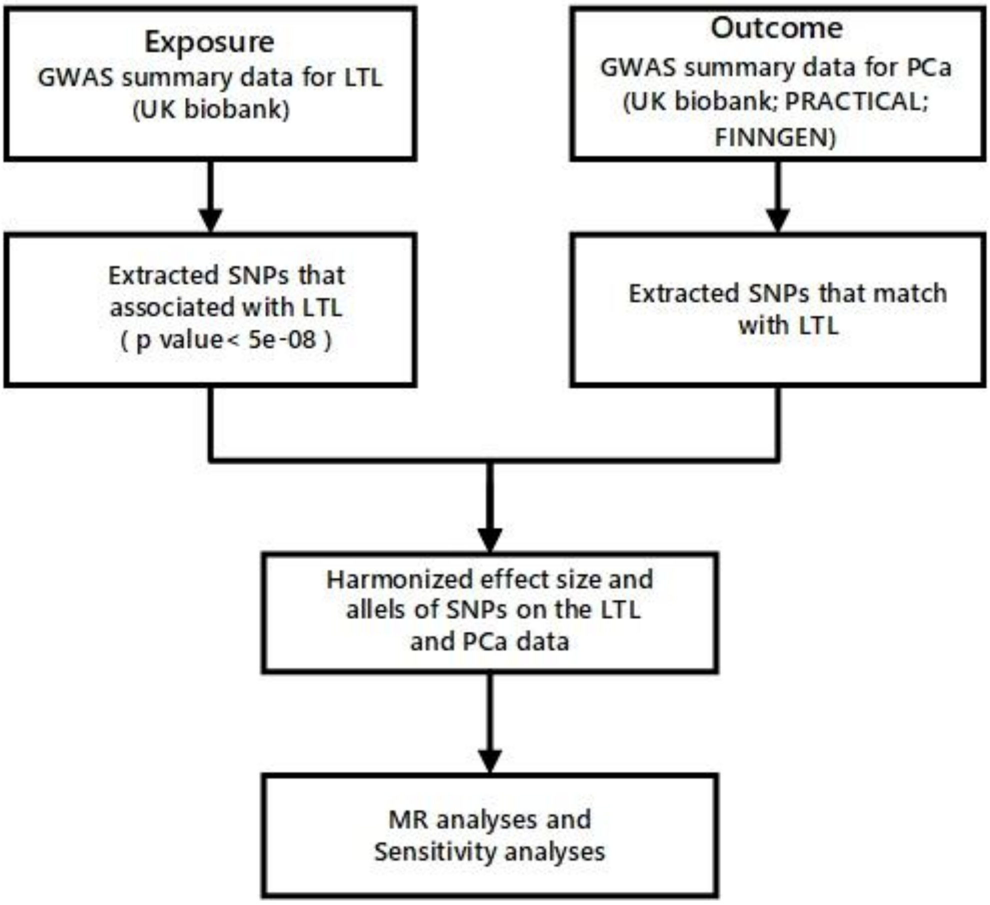

Supplementary Figure S4

Scatter plots illustrating the estimates from Mendelian Randomization (MR) analyses of genetically predicted telomere length and prostate cancer risk; (A) Results for telomere length and prostate cancer from the UK Biobank; (B) Results for telomere length and prostate cancer from the PRACTICAL consortium; (C) Results for telomere length and prostate cancer from the FINNGEN study

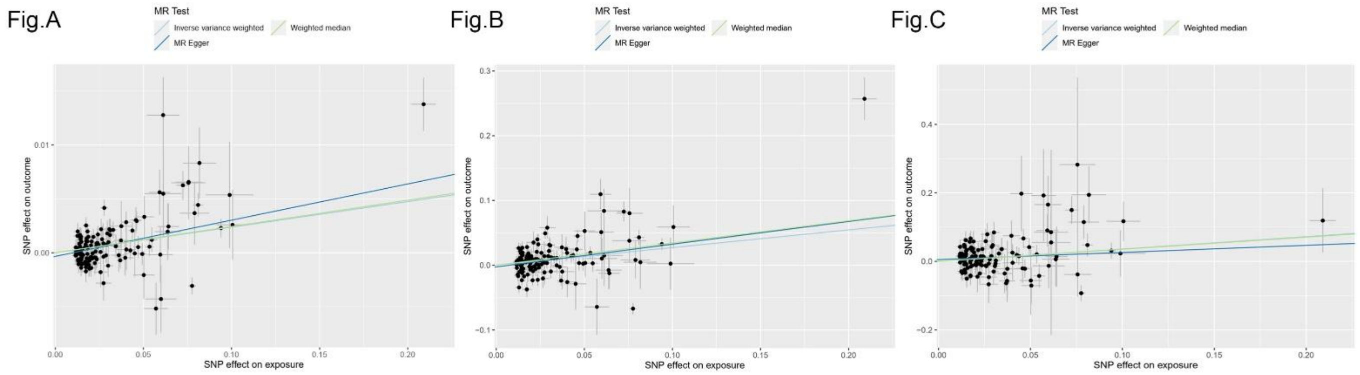

Supplementary Figure S5

Funnel plots of estimates from Mendelian Randomization (MR) analyses of genetically predicted telomere length and prostate cancer risk; (A) Results for telomere length and prostate cancer risk from the UK Biobank; (B) Results for telomere length and prostate cancer risk from the PRACTICAL consortium; (C) Results for telomere length and prostate cancer risk from the Finnish study (FINN)

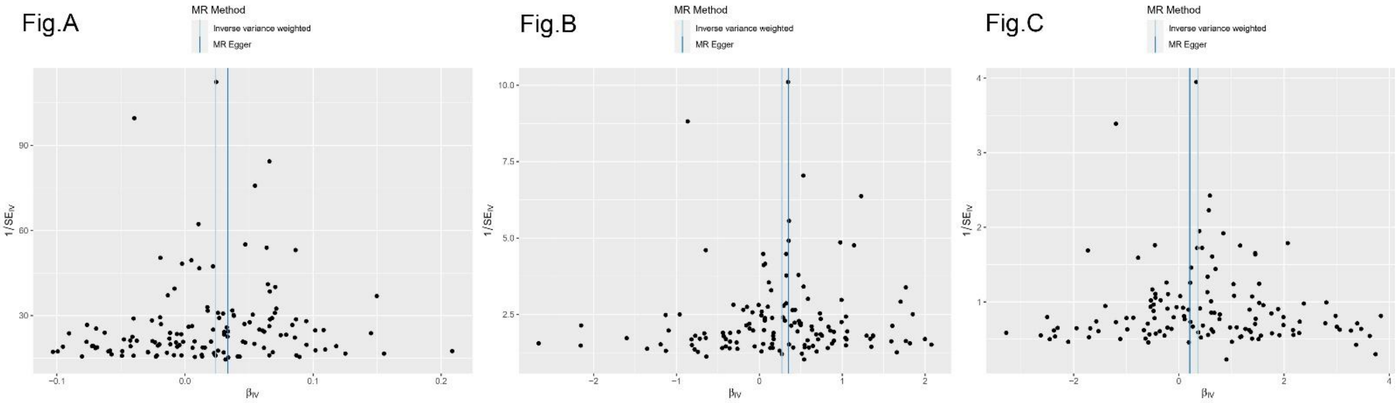

Supplementary Figure S6

Leave-One-Out Sensitivity Analysis for Mendelian Randomization of Genetically Predicted Telomere Length and Prostate Cancer Risk: (A) Results for Telomere Length and Prostate Cancer from the UK Biobank; (B) Results for Telomere Length and Prostate Cancer from PRACTICAL; (C) Results for Telomere Length and Prostate Cancer from FINN study

Fig.A

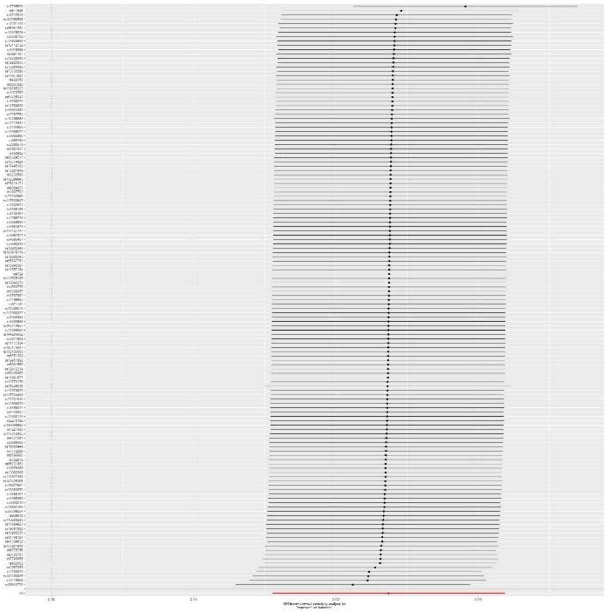

Fig.B

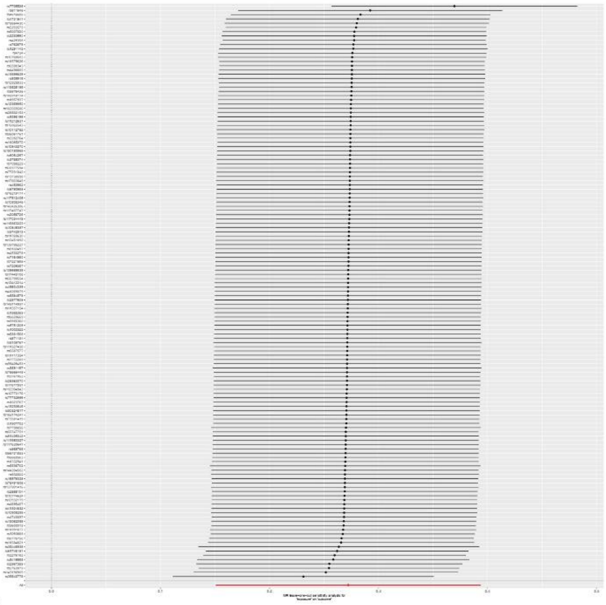

Fig.C

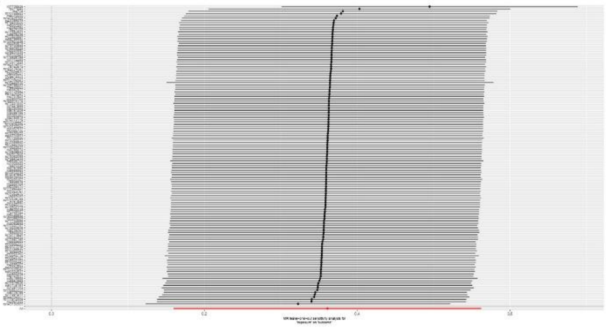

Supplement: Online Supplementary Document [file jogh-15-04228-s001.pdf]
